# Amphiregulin Regulates Phagocytosis-Induced Cell Death in Monocytes via EGFR and the Bcl-2 Protein Family

**DOI:** 10.1155/2019/1603131

**Published:** 2019-11-03

**Authors:** Christopher Platen, Stephan Dreschers, Jessica Wappler, Andreas Ludwig, Stefan Düsterhöft, Lucy Kathleen Reiss, Thorsten W. Orlikowsky

**Affiliations:** ^1^Department of Neonatology, University Children's Hospital, Aachen, Germany; ^2^Molecular Tumor Biology, Department of General, Visceral and Transplantation Surgery, University Hospital, Aachen, Germany; ^3^Institute of Pharmacology and Toxicology, Medical Faculty, RWTH Aachen University, Aachen, Germany

## Abstract

Neonates are extremely susceptible to bacterial infections, and evidences suggest that phagocytosis-induced cell death (PICD) is less frequently triggered in neonatal monocytes than in monocytes from adult donors. An insufficient termination of the inflammatory response, leading to a prolonged survival of neonatal monocytes with ongoing proinflammatory cytokine release, could be associated with the progression of various inflammatory diseases in neonates. Our previous data indicate that amphiregulin (AREG) is increasingly expressed on the cell surface of neonatal monocytes, resulting in remarkably higher soluble AREG levels after proteolytic shedding. In this study, we found that *E. coli*-infected neonatal monocytes show an increased phosphorylation of ERK, increased expression of Bcl-2 and Bcl-X_L_, and reduced levels of cleaved caspase-3 and caspase-9 compared to adult monocytes. In both cell types, additional stimulation with soluble AREG further increased ERK activation and expression of Bcl-2 and Bcl-X_L_ and reduced levels of cleaved caspase-3 and caspase-9 in an EGFR-dependent manner. These data suggest that reduced PICD of neonatal monocytes could be due to reduced intrinsic apoptosis and that AREG can promote protection against PICD. This reduction of the intrinsic apoptosis pathway in neonatal monocytes could be relevant for severely prolonged inflammatory responses of neonates.

## 1. Introduction

Activation of the immune system by bacterial infections induces a biphasic host response in adults. The first, acute inflammatory response is primarily beneficial as a defense mechanism. However, it can also cause tissue injury and lead to various diseases in case the inflammatory response is excessive and prolonged [1]. In order to avoid damage, the immune system needs tight controls and has a variety of mechanisms which actively terminate the reaction [2]. Monocytes are a cell type of the myeloid lineage which play a prominent role in pathogen defense but can also contribute to the establishment of sustained inflammation [3]. These phagocytic cells eliminate microbes and particles via phagocytosis and orchestrate the subsequent immune reaction [4]. Under physiological conditions, their activity is terminated by a special form of apoptosis called phagocytosis-induced cell death (PICD) [5]. A sufficient termination of the inflammation is particularly important for neonates, because evidence suggests that the prolonged inflammation is associated with various pathologies [6–8]. Given the fact that neonatal monocytes obtained from cord blood (CBMO) show considerably decreased PICD compared to monocytes from peripheral blood adult donors (PBMO), even though both feature equal phagocytic and intracellular degradation activities [9], insufficient PICD might be a potential causing factor for prolonged inflammatory diseases of neonates.

In principle, PICD can be triggered by the intrinsic and the extrinsic apoptosis signaling pathways [10, 11]. Activation of the intrinsic pathway, which is also referred to as the mitochondrial apoptosis pathway, is caused by a variety of factors including growth factor starvation, DNA damage, osmotic pressure, or infections [12] and leads to the activation of the Bcl-2 (B-cell lymphoma 2) family member Bax (Bcl-2-associated X). In conjunction with the protein Bak (Bcl-2-antagonist/killer), this proapoptotic protein forms the mitochondrial apoptosis-induced channel (MAC) and activates the mitochondrial permeability transition pore (mPTP) [13, 14]. Activation leads to the loss of the mitochondrial transmembrane potential and finally the release of cytochrome c from the mitochondria [15].

Inside the cytosol, the released cytochrome c can bind Apaf-1 (apoptotic protease activating factor 1) [16], thereby mediating stepwise assembly into a heptameric complex referred to as apoptosome. Binding of the initiator pro-caspase-9 to the complex enables its autocatalytic activation [17]. Active caspase-9 mediates activation of caspase-3, finally leading to apoptosis [18].

Formation of MAC can be prevented by binding of the antiapoptotic proteins Bcl-2 and/or Bcl-X_L_ to Bax [19–21]. In turn, this protective mechanism can be neutralized by the protein BAD (Bcl-2-antagonist of cell death). BAD can heterodimerize with Bcl-X_L_ or Bcl-2 and can thereby promote cell death [22]. However, BAD proapoptotic activity is controlled via its phosphorylation status. Protein kinases can phosphorylate BAD, which leads to the dissociation of the complex and thereby enables Bcl-2/Bcl-X_L_ to inhibit Bax-induced apoptosis [23]. The group of protein kinases that mediate phosphorylation of BAD includes Akt, regulated via the PI3K (phosphoinositide 3-kinase)/Akt signaling pathway [24], as well as ERK1/2, regulated via MAPK (mitogen-activated protein kinase)/ERK (extracellular signal-regulated kinase) pathway and p90^RSK^ [25, 26]. Interestingly, both the PI3K/Akt and the MAPK/ERK pathway are activated by EGFR (epidermal growth factor receptor), a member of the tyrosine kinase receptor family [27–29]. EGFR is a broadly expressed receptor on epithelial cells but has also been found on monocytes [30, 31].

One well-known EGFR ligand is amphiregulin (AREG) [32]. AREG is a growth factor of the EGF family [33], which was shown to be expressed on monocytes [34] and T cells [35] and to control the inflammation process [36]. Synthesized as the transmembrane precursor pro-AREG, the soluble protein is released by limited proteolytic cleavage of the precursor [37]. In a previous study, we demonstrated that CBMO have a higher pro-AREG surface expression compared to PBMO. Hence, upon *E. coli* infection-induced cleavage of pro-AREG, CBMO show an 11-fold higher level of soluble AREG compared to PBMO [38]. We further showed that AREG increases intracellular MMP-2 and MMP-9 levels and induces cleavage of membrane-bound FasL through engagement with the EGF receptor, pointing towards involvement of the extrinsic apoptosis pathway. Reduction of AREG levels was found to diminish PICD in CBMO and PBMO [38].

Due to the incomplete reduction of PICD by the FAS/FASL system, we hypothesize that the differences in pro-AREG expression and shedding could also affect the intrinsic apoptosis signaling. Given that insufficient termination of the inflammatory response in neonates involves the risk of severe sequelae, we sought to investigate whether AREG could be targeted to initiate PICD in neonates. Our findings indicate that AREG can prevent intrinsic apoptosis pathways in neonatal monocytes supporting the concept that it may serve as a potential target for prevention of prolonged inflammation in neonates.

## 2. Material and Methods

### 2.1. Patient Samples

The presented experimental procedure was approved by the Ethics Committee of Aachen University Hospital (Permission No: EK150/09, Oct. 6, 2009). All adult participants gave written informed consent prior to have taken their venous blood samples. Solely neonates, which were delivered spontaneously and did not show signs of infection, were accepted for this study. Health status was determined by examination of white blood cell count, Interleukin-6 (IL-6), C-reactive protein, and the clinical status. Immediately after cord ligation, umbilical cord blood samples were placed in heparin-coated tubes (10 IU/ml blood). Mothers showing either amnion infection or prolonged (>12 hours) labor as well as SGA neonates (small for gestational age) and preterm infants before 36 weeks of gestation were not accepted for this study.

### 2.2. Mononuclear Cell Culture

By using Ficoll density gradient centrifugation (Amersham, Freiburg, Germany), human cord blood mononuclear cells (CBMC) as well as human peripheral blood mononuclear cells (PBMC) were isolated. Afterwards, the cells were washed with PBS, and monocytes were separated from remaining cell types by using the magnetic cell sorting monocyte isolation kit II (Miltenyi Biotec, Bergisch Gladbach, Germany) according to the manufacturer's recommendation. Detected by flow cytometry, the method routinely yielded 95% purity of the population while 90% CD14-positive cells were defined as minimal cut-off value. The cells were standardly cultivated in VLE RPMI-1640 medium (Biochrom, Berlin, Germany) containing 10% heat-inactivated fetal bovine serum (FBS, Biochrom, Germany) and 1% penicillin/streptomycin (Thermo Fisher, Massachusetts, USA). Postphagocytic reaction experiments were performed in 24-well cell culture plates (Costar, Bodenheim, Germany) containing 1 × 10^6^ monocytes/ml.

### 2.3. Bacterial Culture

Two bacterium strains were used for phagocytosis experiments. *E. coli* dsRed (strain BL21) were a kind gift from Prof. L. Rink (Institute of Immunology, RWTH Aachen University, Germany) [39]. This strain is transformed with the vector pGEX-4T-1, containing the gene for the recombinant red fluorescent protein (dsRed). *E. coli* DH5*α* is an encapsulated K12 laboratory strain. Bacteria were standardly grown in Lennox-L-Broth-medium (Thermo Fisher, Massachusetts, USA) until early logarithmic phase and were then immediately harvested. Human monocytes were infected as follows: 1 × 10^6^ PBMC/CBMC per ml were incubated for 1 h with *E. coli* DH5*α* or *E. coli* dsRed in culture medium without antibiotics at a multiplicity of infection (MOI) of 20 : 1. Afterwards, extracellular bacteria were removed by washing the cells with FBS. The infected cells were cultivated for another 23 h to accomplish an overall cultivation time of 24 h after infection before analyzing postphagocytic reaction.

### 2.4. Stimulation and Inhibitor Treatment

For monocyte stimulation, recombinant human AREG obtained from R&D Systems (Minneapolis, USA) was aliquoted in sterile PBS with 0.1% BSA and then used in a final concentration of 0.5 or 0.05 *μ*g/ml. For comparison, monocytes were stimulated with human recombinant 0.1 *μ*g/ml EGF (Sigma, Taufkirchen, Germany). Neutralizing EGFR antibody (clone D1D4J) was purchased from Cell Signaling (Frankfurt, Germany) and was applied in a final dilution of 1 : 100. Stimulation and inhibitor treatment were started 1 h prior to infection and were continued during the time of experimental cultivation. Cells treated with gefitinib were used as a control. Gefitinib (Selleckchem, Munich, Germany), which inhibits the tyrosine kinase portion of the EGFR, were used as a control. Therefore, cells were treated simultaneously with *E. coli* and 5 ng/ml gefitinib. After 1 h, bacteria were removed and gefitinib treatment was continued for 23 h.

Isolation of the cytosolic fraction of monocytes was performed by resuspending 5 × 10^6^ treated and nontreated monocytes with 300 *μ*l PBS containing a protease inhibitor cocktail (Carl Roth GmbH, Karlsruhe, Germany) and subjecting the cells to three rounds of freeze-thaw cycles. Afterwards, the crude cell lysate was centrifuged two times at 4°C. The 4-time concentrated Laemmli buffer was added to the supernatants. After boiling for three minutes, the lysates were deployed to SDS-PAGE (according to standard protocols of Lämmli and Khyse Anderson). For imaging and quantification, a LAS 3000 imager (Fujifilm, Düsseldorf, Germany) combined with the Multi-Gauge software (Fujifilm, Düsseldorf, Germany) was used.

### 2.5. Analysis of Intracellular Antigens

Expression of antigens was analyzed by using a FACSCanto flow cytometer (Becton Dickinson, Mountain View, USA). Gating was performed by using the expression of CD14, forward scatter (FSC), and sideward scatter (SSC). For the analysis of intracellular antigens, monocytes were permeabilized by applying Perm/Wash buffer purchased from Becton Dickinson Biosciences (Franklin Lakes, New Jersey, USA) for 20 min at RT. Antibodies and the corresponding isotype control were diluted in Perm/Wash buffer according to the manufacturer's recommendation and incubated for 1 h at 4°C. Finally, the cells were washed with PBS containing 0.1% BSA, before they were analyzed. For detection on immunoblots, the cytochrome c antibody, purchased from BD Pharmingen (San Jose, USA; clone 7H8.2C12), was used.

### 2.6. Antibodies

Antibodies against phospho-p44/42 MAPK (pERK1, Thr202/Tyr204, pERK2, T185/Y187, and polyclonal rabbit IgG), phospho-P38 (Thr180/Tyr182, clone D3F9), P38 (clone D13E1), phospho-JNK-1/-2 (Thr183/Tyr185, clone G9), JNK-1/-2, pAKT (Ser473, polyclonal rabbit IgG), cleaved caspase-3 (Asp175, clone 5A1E), and cleaved caspase-9 (Asp330, clone D2D4) were obtained from BD Pharmingen (San Jose, USA). The Bcl-2 (C-2, clone AF647) antibody was purchased from Santa Cruz Biotechnology (Dallas, USA), and the corresponding FITC-labeled anti-mouse IgG1 (clone 1F8) secondary antibody was purchased from ImmunoTools (Friesoythe, Germany). PE-labeled pBAD (Ser112, clone 40A9) antibody, MAPK detecting antibodies, and corresponding rabbit IgG isotype control (clone DA1E) were obtained from Cell Signaling (Frankfurt, Germany). For detection of CD14, either an APC-labeled CD14 antibody (clone MEM-15) or a FITC-labeled CD14 antibody (clone MEM-18), both purchased from ImmunoTools (Friesoythe, Germany), was used. Alexa Fluor 488-labeled Bcl-X_L_ (clone 54H6) antibody was purchased from Cell Signaling (Frankfurt, Germany), and the corresponding rabbit IgG isotype control (clone 60024B) was purchased from R&D Systems (Minneapolis, USA). Staining procedures were performed according to the manufacturer's recommendation.

### 2.7. Statistical Analysis

For all analyses, GraphPad Prism 7 statistical software (GraphPad Software, La Jolla, USA) was used. Data are presented as means + standard deviation (SD) and were statistically analyzed using either one-way ANOVA or two-way ANOVA with Bonferroni's multiple comparisons test. Values of *p* < 0.05 were considered as statistically significant. Where indicated, Student's *t*-test with Welch correction was performed.

## 3. Results

### 3.1. AREG Mediates Phosphorylation of ERK1/2 and Akt

We had previously demonstrated that AREG regulates matrix metalloprotease-2- and matrix metalloprotease-9-mediated extrinsic FAS-mediated apoptosis via activation of EGFR downstream signaling [38]. Since EGFR is also capable of mediating intrinsic apoptosis through MAPK (ERK, p38, and JNK) and PI3K/Akt signaling [27–29], we wanted to examine whether AREG affects intrinsic apoptosis in PBMO and CBMO through EGFR.

In the first step, we investigated whether infection with *E. coli* or AREG stimulation would affect the number of EGFR-positive cells in our PBMO and CBMO populations. As previously reported, infection with *E. coli* increased the number of EGFR-expressing PBMO and CBMO [38]. By contrast stimulation with AREG did not show a significant effect (Table 1). This is in line with previous results showing that EGFR density (MFI) significantly increases after infection with *E. coli* [38].

**Figure 1:**
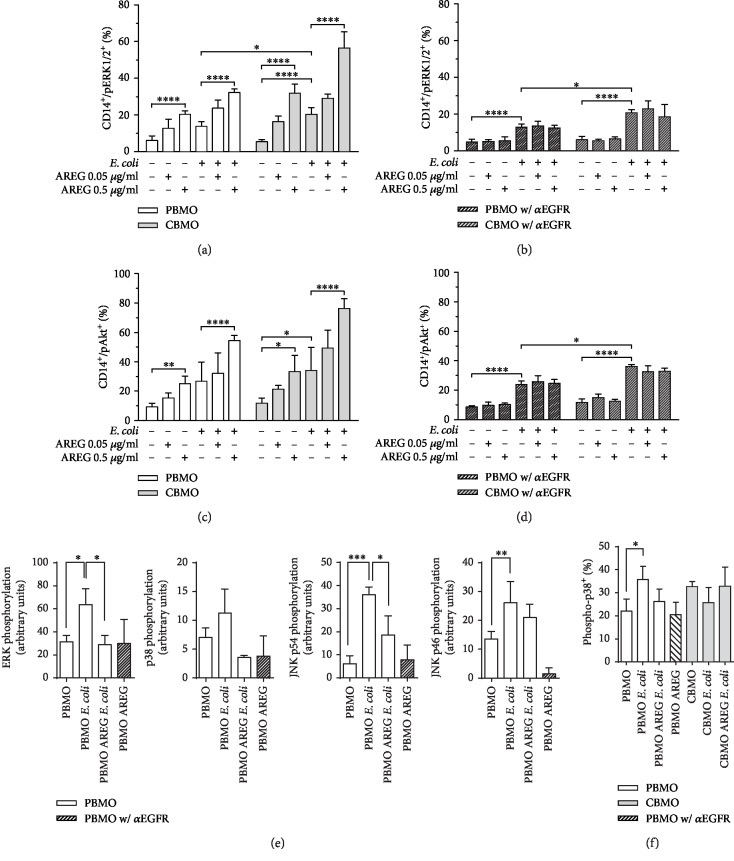
MAPK and Akt phosphorylation—levels of activated monocytes and intracellular protein levels in PBMO and CMBO in response to infection, AREG stimulation, and inhibition of EGFR. Isolated monocytes were infected with *E. coli*, extracellular bacteria were removed, and the cells were cultivated for 24 h in medium supplemented with antibiotics. 1 h prior to infection, AREG stimulation and EGFR inhibitor treatment were started and maintained during the entire cultivation period. Quantification was performed by flow cytometry (a–d, f). (a) Detection of pERK1/2 in PBMO and CBMO in response to infection and AREG stimulation. AREG stimulation dose-dependently induces phosphorylation in all settings. ERK1/2 phosphorylation is induced in response to *E. coli* infection (*n* = 5). (b) Effect of EGFR inhibition on pERK1/2 in PBMO and CBMO in response to infection and AREG stimulation. Inhibition of EGFR abolishes the AREG-dependent phosphorylation (*n* = 4). (c) Detection of pAkt in PBMO and CBMO in response to infection and AREG stimulation. AREG stimulation dose-dependently increases pAkt levels in all settings. Akt phosphorylation is induced in response to *E. coli* infection (*n* = 5). (d) Effect of EGFR inhibition on pAkt in PBMO and CBMO in response to infection and AREG stimulation. Inhibition of EGFR abolishes the AREG-dependent phosphorylation (*n* = 4). Quantification of immunoblot signals on which the indicated MAPK were detected ((e); *n* = 3). Detection of pp38 in PBMO and CBMO in response to infection and AREG stimulation ((f); *n* = 4). Data are shown as means + SD. Statistical significance was analyzed using two-way ANOVA with Bonferroni's multiple comparisons test (^∗^*p* < 0.05; ^∗∗^*p* < 0.01; and ^∗∗∗∗^*p* < 0.001). In (e, f), error bars detail results of unpaired Student's *t*-test with Welch correction (^∗^*p* < 0.05; ^∗∗∗^*p* < 0.005).

Next, we investigated whether AREG induces EGFR-mediated phosphorylation of ERK1/2 and Akt in PBMO and CBMO. ERK1/2 phosphorylation was significantly induced in response to *E. coli* infection (Figure 1(a)). Additionally, we found that AREG dose-dependently induces phosphorylation in both control and *E. coli*-infected PBMO and CBMO. Interestingly, in each setting, pERK1/2 levels were significantly higher in CBMO as compared to PBMO (Figure 1(a)). Furthermore, inhibition of EGFR abolished the phosphorylation-inducing effect of AREG (Figure 1(b)), suggesting that ERK1/2 phosphorylation is attributable to AREG-mediated activation of EGFR.

Just like pERK1/2, pAkt levels were significantly increased in response to *E. coli* infection. In both control and *E. coli*-infected cells, Akt phosphorylation was dose-dependently enhanced in response to AREG stimulation. In each setting, pAkt levels were significantly higher in CBMO than in PBMO (Figure 1(c)). The AREG dose-dependent increase in pAkt was abolished by inhibition of EGFR, suggesting that AREG does induce phosphorylation not only of ERK1/2 but also of Akt via EGFR signaling (Figure 1(d)). Noteworthily, EGFR inhibition did abolish neither infection-induced ERK1/2 nor Akt phosphorylation, suggesting that infection may initiate another signaling pathway which phosphorylates both proteins independently of EGFR.

To address the question, whether other MAPK beside ERK1/2 are activated after *E. coli* infection, we compared the phosphorylation pattern of JNK1/2 and P38 in infected PBMO via immunoblot analysis (Figure 1(e), Supplementary [Supplementary-material supplementary-material-1]). In line with the results of the FACS analysis, ERK1/2 was found phosphorylated in response to *E. coli* infection (Figure 1(e), A). Furthermore, ERK1/2 phosphorylation could be shown to be reduced by AREG. In contrast, p38 did not exhibit a significant activation after *E. coli* infection (Figure 1(e), B). Both splice variants of JNK1/2 were found phosphorylated after infection, but only the higher molecular weight variant p54 was found less phosphorylated after AREG treatment (Figure 1(e), C and D). CBMO did not show significant levels of phosphorylated JNK (*p* > 0.5, infected CBMO vs. control).

For p38, we further confirmed the obtained results by performing a FACS-based assay. Here, p38 showed a specific phosphorylation after *E. coli* infection, but a combined treatment with AREG and *E. coli* failed to reduce the phosphorylation (Figure 1(f)). Additionally, we found no significant phosphorylated p38 in *E. coli*-infected CBMO (Figure 1(f)).

### 3.2. AREG Leads to Phosphorylation of BAD

**Figure 2:**
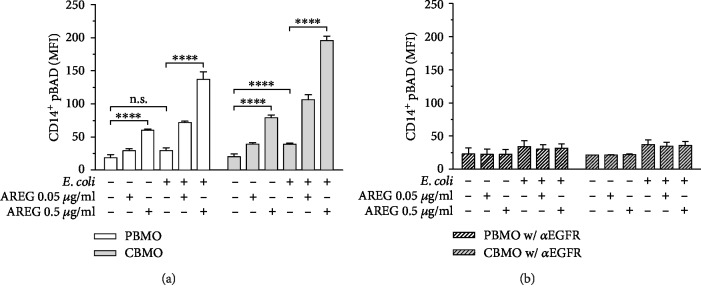
pBAD intracellular protein levels in PBMO and CMBO in response to infection, AREG stimulation, and inhibition of EGFR. Isolated monocytes were infected with *E. coli*, extracellular bacteria were removed, and the cells were cultivated for 24 h in medium supplemented with antibiotics. 1 h prior to infection, AREG stimulation and EGFR inhibitor treatment were started and maintained during the entire cultivation period. Quantification was performed by using flow cytometry. (a) pBAD in PBMO and CBMO in response to infection and AREG stimulation. *E. coli* infection leads to BAD phosphorylation. Increase in pBAD is dose-dependently induced by AREG in both control and *E. coli*-infected PBMO and CBMO (*n* = 5). (b) Effect of EGFR inhibition on pBAD in PBMO and CBMO in response to infection and AREG stimulation. Inhibition of EGFR abolishes both AREG- and infection-induced increase in pBAD levels (*n* = 4). Data are shown as means + SD. Statistical significance was analyzed by using two-way ANOVA with Bonferroni's multiple comparisons test. n.s.: not significant. ^∗∗∗∗^*p* < 0.001.

Since we demonstrated that both ERK1/2 and Akt phosphorylations are induced by infection-mediated activation of EGFR, we wanted to investigate whether these signaling events mediate phosphorylation of the protein BAD through AREG signaling. Matching the previous results, *E. coli* infection did only lead to BAD phosphorylation in CBMO but not in PBMO. Furthermore, the increase in pBAD was dose-dependently enhanced by AREG stimulation in both control and *E. coli*-infected cells (Figure 2(a)). Inhibition of EGFR abolished both AREG- and infection-induced increases in pBAD (Figure 2(b)), suggesting that phosphorylation of BAD is induced by EGFR-mediated signaling.

### 3.3. AREG Increases Intracellular Bcl-2 and Bcl-X_L_ Levels

Phosphorylation of BAD leads to dissociation of the BAD/Bcl-2 and the BAD/Bcl-X_L_ heterodimer, respectively, resulting in free Bcl-2 and Bcl-X_L_ which play a critical antiapoptotic role [19, 21]. Therefore, the next step was to detect whether the demonstrated AREG-mediated increase in pBAD indeed causes intracellular accumulation of unbound Bcl-2 and Bcl-X_L_.

**Figure 3:**
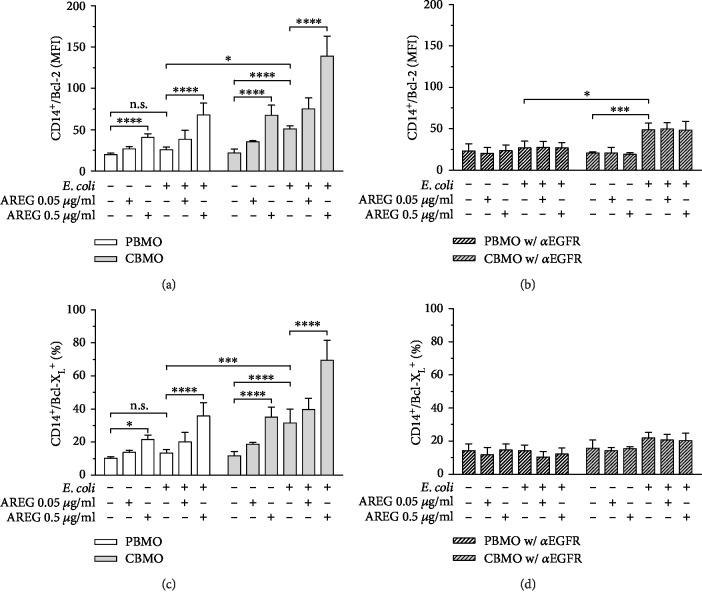
Bcl-2 and Bcl-X_L_ intracellular protein levels in PBMO and CMBO in response to infection, AREG stimulation, and inhibition of EGFR. Isolated monocytes were infected with *E. coli*, extracellular bacteria were removed, and the cells were cultivated for 24 h in medium supplemented with antibiotics. 1 h prior to infection, AREG stimulation and EGFR inhibitor treatment were started and maintained during the entire cultivation period. Quantification was performed by using flow cytometry. (a) Bcl-2 in PBMO and CBMO in response to infection and AREG stimulation. Bcl-2 levels are dose-dependently induced upon AREG stimulation in all settings. *E. coli* infection leads to an increase in Bcl-2 levels in CBMO but not in PBMO (*n* = 5). (b) Effect of EGFR inhibition on Bcl-2 in PBMO and CBMO in response to infection and AREG stimulation. AREG-mediated but not infection-mediated increase in Bcl-2 levels is suppressed by EGFR inhibition (*n* = 4). (c) Bcl-X_L_ in PBMO and CBMO in response to infection and AREG stimulation. AREG stimulation dose-dependently increases Bcl-X_L_ levels in both groups, while *E. coli* infection increases Bcl-X_L_ solely in CBMO (*n* = 5). (d) Effect of EGFR inhibition on Bcl-X_L_ levels in PBMO and CBMO in response to infection and AREG stimulation. AREG and infection-mediated increase in Bcl-X_L_ levels is abolished by EGFR inhibition (*n* = 4). Data are shown as means + SD. Statistical significance was analyzed using two-way ANOVA with Bonferroni's multiple comparisons test. n.s.: not significant. ^∗^*p* < 0.05; ^∗∗∗^*p* < 0.005; and ^∗∗∗∗^*p* < 0.001.

Bcl-2 levels were dose-dependently increased in response to AREG stimulation in both control and *E. coli*-infected PBMO and CBMO. *E. coli* infection alone led to an increase in Bcl-2 only in CBMO but not in PBMO (Figure 3(a)). In PBMO, inhibition of EGFR abolished both AREG- and infection-induced increases in intracellular Bcl-2 levels; however, in CBMO, it did only suppress the increase which was mediated by stimulation with AREG (Figure 3(b)). When analyzing intracellular Bcl-X_L_ levels in response to infection and AREG stimulation, we observed a similar induction as seen for Bcl-2 (Figure 3(c)). In contrast to Bcl-2, the increase in Bcl-X_L_ in response to both AREG and *E. coli* infection was abolished by EGFR inhibition (Figure 3(d)).

### 3.4. AREG Reduces Caspase-9 and Caspase-3 Cleavage after *E. coli* Infection

**Figure 4:**
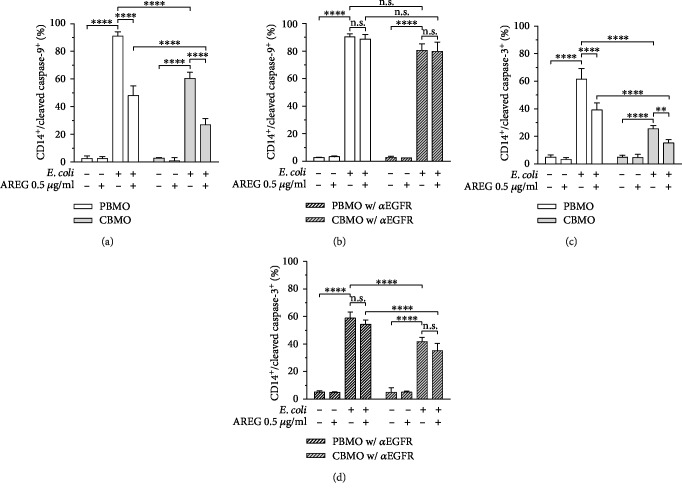
Intracellular levels of cleaved caspase-9 and cleaved caspase-3 in PBMO and CMBO in response to infection, AREG stimulation, and inhibition of EGFR. Isolated monocytes were infected with *E. coli*, extracellular bacteria were removed, and the cells were cultivated for 24 h in medium supplemented with antibiotics. 1 h prior to infection, AREG stimulation and EGFR inhibitor treatment were started and maintained during the entire cultivation period. Detection was performed by flow cytometry. (a) Cleaved caspase-9 in PBMO and CBMO in response to infection and AREG stimulation. In both PBMO and CBMO, cleaved caspase-9 levels were significantly increased in response to *E. coli* infection, whereas AREG stimulation in turn leads to a decrease in cleaved caspase-9 levels (*n* = 5). (b) Effect of EGFR inhibition on cleaved caspase-9 levels in PBMO and CBMO in response to infection and AREG stimulation. EGFR inhibition abolishes the AREG-mediated decrease in cleaved caspase-9 in both infected PBMO and CBMO (*n* = 4). (c) Cleaved caspase-3 in PBMO and CBMO in response to infection and AREG stimulation. In both groups, cleaved caspase-9 levels were significantly increased in response to *E. coli* infection, while AREG stimulation diminishes the effect (*n* = 5). (d) Effect of EGFR inhibition on cleaved caspase-3 in PBMO and CBMO in response to infection and AREG stimulation. In both infected PBMO and CBMO, EGFR inhibition offsets the AREG-mediated decrease in cleaved caspase-3 levels (*n* = 4). Data are shown as means + SD. Statistical significance was analyzed using two-way ANOVA with Bonferroni's multiple comparisons test. n.s.: not significant. ^∗∗^*p* < 0.01; ^∗∗∗∗^*p* < 0.001.

AREG-induced induction of Bcl-2 and Bcl-X_L_ should lead to increased Bcl-2 and Bcl-X_L_ binding to Bax and thereby prevent the formation of MAC. MAC function is known to be critical for the intrinsic signaling pathway resulting in formation of the active apoptosome complex which mediates activation of caspase-3, finally leading to apoptosis [18]. Hence, activity of the intrinsic apoptosis pathway can be detected by measuring the cleavage of the caspases. Therefore, we measured both cleaved caspase-9 and caspase-3 in response to *E. coli* infection and phagocytosis of *E. coli* and investigated whether AREG stimulation is capable of inhibiting the cleavage by preventing the formation of MAC via EGFR. In response to *E. coli* infection, cleaved caspase-9 levels were significantly increased, indicating phagocytosis-induced cell death by apoptosis as it was expected. Importantly, the infection-mediated increase in the cleaved caspase-9 level was clearly higher in PBMO than in CBMO confirming that CBMO have a reduced potential to undergo intrinsic apoptosis when compared to PBMO (Figure 4(a)). Moreover, AREG stimulation significantly decreased cleaved caspase-9 levels in both infected PBMO and infected CBMO. Of note, AREG did not affect basal levels of cleaved caspase-9 in uninfected cells (Figure 4(a)). Inhibition of EGFR abolished the AREG-mediated decrease in cleaved caspase-9 in both infected PBMO and CBMO (Figure 4(b)). Furthermore, the clear reduction of cleaved caspase-9 in CBMO compared to PBMO (Figure 4(a)) is abolished when EGFR is blocked (Figure 4(b)). When measuring cleaved caspase-3 in response to infection and AREG stimulation, we obtained similar results as for cleaved caspase-9 (Figure 4(c)). Again, the infection-mediated increase in the cleaved caspase-3 level was clearly higher in PBMO than in CBMO, and inhibition of EGFR increased cleaved caspase-3 levels in infected CBMO but not in PBMO. In addition, in both infected PBMO and CBMO, EGFR inhibition prevented the AREG-mediated decrease in cleaved caspase-3 levels (Figure 4(d)). This is in line with the results regarding cleaved caspase-9, indicating that AREG is capable of regulating intrinsic apoptosis in monocytes via EGFR signaling. The increase of caspase-3 cleavage by blocking EGFR was lower (10%) compared to the increase of caspase-9 cleavage by the same treatment (20%; Figures 4(c) and 4(d)).

### 3.5. EGFR Activation Reduces Hypodiploid Cell Formation and Release of Cytochrome c after *E. coli* Infection

**Figure 5:**
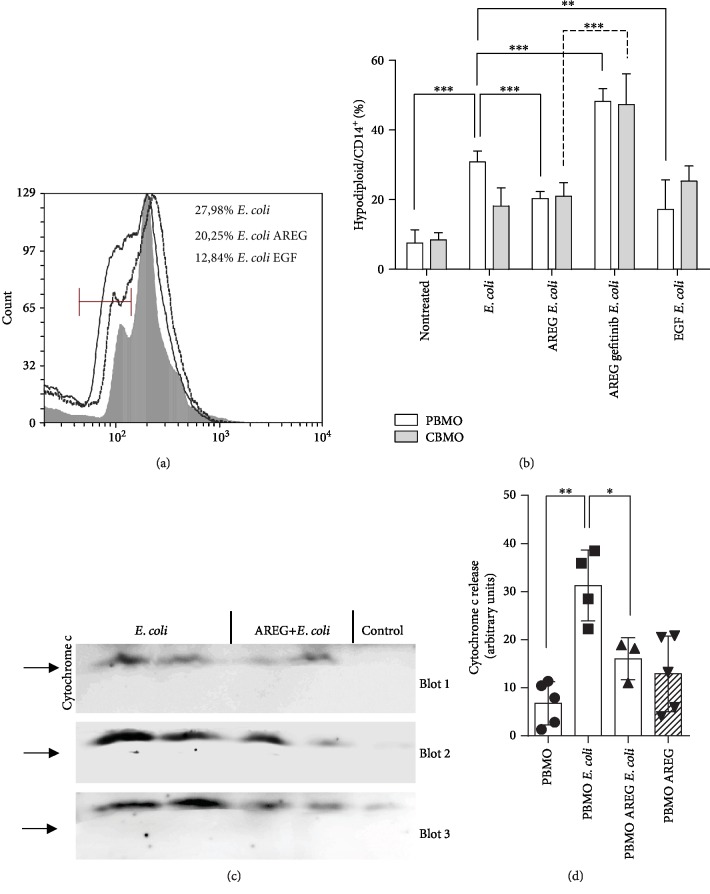
Quantification of PICD via detection of hypodiploid nuclei in PBMO and CBMO. Representative histogram of apoptotic sub-G1 subpopulations of treated PBMO (a; full line: *E. coli*, dotted line: AREG and *E. coli*, and grey area: EGF and *E. coli*). Statistical analysis of the Nicoletti assay 24 h p.i. (statistical analysis using nonpaired Student's *t*-test with Welch correction; ^∗∗^*p* < 0.01; ^∗∗∗^*p* < 0.005). Immunoblots representing the cytochrome c release after indicated treatment of PBMO (c). Statistical analysis of cytochrome c release (d; nonpaired Student's *t*-test with Welch correction; ^∗^*p* < 0.05; ^∗∗^*p* < 0.001).

As an indicator of apoptosis, we next measured hypodiploid cell formation. Proving earlier results [38], treatment with AREG reduced PICD of PBMO after *E. coli* infection (Figures 5(a) and 5(b)). To support this finding, pharmaceutical EGFR blockage by gefitinib was performed. Gefitinib suppressed AREG-driven reaction in PBMO and CBMO and almost doubled the PICD (Figure 5(b)).

Furthermore, we compared the antiapoptotic function of AREG with that of EGF, which also binds to EGFR (Figures 5(a) and 5(b)). The induction of apoptosis in *E. coli*-infected PBMO could be reduced by EGF to the same extent as shown for AREG (Figure 5(a) and compare with Figure 5(b), 4^th^ columns and 5^th^ columns).

To finally test whether gatekeeping proteins of the intrinsic apoptotic pathway are functionally active, we quantified the release of cytochrome c (Figures 5(b) and 5(c)). The infection with *E. coli* induced a strong cytochrome c release in PBMO compared to the uninfected control. The preincubation of infected cells with AREG reduced the cytochrome c efflux about 50% (Figure 5(d), compare second and third columns). Addition of AREG to uninfected cells had no effect. CBMO exhibited no significant cytochrome c release after infection (5.9 ± 7.5 vs. 0.2 ± 0.35 of nontreated CBMO, *p* > 0.05, data not shown).

**Figure 6:**
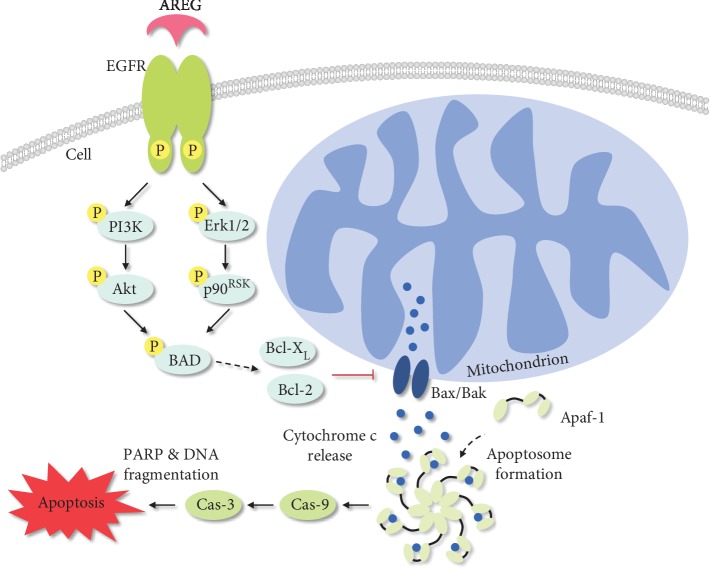
Schematic diagram displaying the impact of AREG on intrinsic apoptosis signaling. Phagocytosis of bacterial pathogens leads to the release of cytochrome c from the mitochondria through the mitochondrial apoptosis-induced channel (MAC), which is formed by the proteins Bax and Bak. In complex with Apaf-1, the released cytochrome c forms the apoptosome, which finally activates caspase-3 through binding and activation of caspase-9. Caspase-3 finally leads to the initiation of apoptosis. The signaling pathway can be inhibited by AREG-mediated activation of the EGF receptor. Binding of the cytokine AREG leads to an EGFR-mediated activation of the PI3K/Akt and MAPK/ERK pathway, which results in the phosphorylation of the protein BAD. In consequence, the heterodimers of BAD/Bcl-X_L_ and/or BAD/Bcl-2 dissociate, thereby enabling Bcl-2 and Bcl-X_L_ to inhibit Bax-induced apoptosis.

In summary, the demonstrated results indicate that soluble AREG diminishes PICD of monocytes via EGFR-mediated inhibition of the intrinsic apoptosis pathway. Over all, infected CBMO show an increased phosphorylation of ERK, increased Bcl-2 and Bcl-X_L_ levels, and reduced levels of cleaved caspase-3 and caspase-9 compared to PBMO. In both cell types, stimulation with soluble AREG further increases ERK activation as well as levels of Bcl-2 and Bcl-X_L_ and reduces levels of cleaved caspase-3 and caspase-9 in an EGFR-dependent manner (Figure 6).

## 4. Discussion

In the present study, we were able to validate that PICD is less frequently triggered in neonatal monocytes than in monocytes from adult donors [9]. AREG has been investigated extensively for its role in oncogenic processes since it was shown to contribute to many of the “hallmarks of cancer” such as metastasis and angiogenesis [40]. Although AREG is expressed on monocytes [34] and is well known for mediating apoptosis resistance in various cancer cell types [41, 42], to our knowledge, there are no data focusing on the role of AREG in PICD of monocytes. Therefore, we hypothesized that AREG might be a key factor involved in the diminished PICD of neonatal monocytes by inhibiting the intrinsic apoptosis pathway.

It was already demonstrated that AREG is expressed in response to viral [43] and bacterial infections [44]. Previous data of our group suggest that pro-AREG is increasingly expressed on neonatal monocytes, leading to remarkably higher soluble AREG levels after infection compared to monocytes from adults [38]. These findings suggest that AREG may be involved in the prevention of PICD of CBMO. AREG was demonstrated to induce phosphorylation of ERK1/2 in keratinocytes [45] and pancreatic cancer cells [46] as well as Akt in several cancer cell types [46, 47]. Here, we were able to show for the first time that the cytokine induces both ERK1/2 and Akt phosphorylations in monocytes. Our results prove that the effect can be attributed to AREG-mediated EGFR downstream trafficking and activation of the MAPK/ERK and the PI3K/Akt pathway, respectively [27–29]. Furthermore, our results suggest that PICD is inhibited by the prevention of MAC formation and caspase cleavage, mainly through inhibition of the protein Bax by unbound Bcl-2 and Bcl-X_L_. This is in line with our finding that cytochrome c release is diminished by AREG preincubated, *E. coli*-infected PBMO. Indeed, several studies have demonstrated that intrinsic apoptosis is mediated through EGFR signaling. Inhibition of EGFR led to apoptosis by Bax activation [48, 49] or altered the balance of Bax and Bcl-2 expression [50, 51]. Moreover, studies in non-small-cell lung cancer cells already revealed Bax as the connecting element in AREG-induced apoptosis inhibition [26, 42]. Noteworthily, it is not proven whether the antibodies against Bcl-2 and Bcl-X_L_, which we used in our study, exclusively detect the unbound proteins or additionally detect the proteins in complex with BAD. However, since we demonstrated increased pBAD levels, we conclude that the increase in Bcl-2 and Bcl-X_L_ might represent an actual increase in the unbound proteins. Moreover, on the transcription level, it was demonstrated that AREG-dependent EGFR activation reduced Bax expression but enhanced the expression of Bcl-2 and Bcl-X_L_. *Vice versa*, inhibition of EGFR increased Bax and decreased Bcl-2 expression [52]. Future work sought to focus on the question, whether AREG leads to an increased release of Bcl-2 and Bcl-X_L_ from the BAD heterodimer or to an enhanced Bcl-2 and Bcl-X_L_ expression or both.

Additionally, it has to be mentioned that, besides ERK and AKT pathways, other signal transduction pathways can activate the antiapoptotic effect of small Bcl-like proteins [53]. Activation of the JNK MAP kinase after *E. coli* infection could be explained by phagocytosis-dependent ROS formation. However, CBMO, which, compared to PBMO, equally phagocytose *E. coli*, do not exhibit JNK phosphorylation. Further studies have to elucidate the role of JNK signaling, because JNK p46 phosphorylation is not reduced by AREG. A redundant MAPK signaling was also reported for tumor differentiation in EGFR-tyrosine-kinase-resistant cells upon gefitinib treatment [54]. Furthermore, the AREG/EGFR-activated glycogen-synthase-kinase-3-*β* (GSK-3*β*) has recently been described to act in this manner [55].

Bax activity leads to the activation of multiple apoptotic caspases [56], and its activity was shown to be preventable by AREG-mediated activation of EGFR signaling [26, 42]. Our results show that the infection-mediated increase in cleaved caspase-9 and caspase-3 levels is overall higher in PBMO, whereas exclusively in CBMO, EGFR inhibition increases the infection-mediated cleavage of both caspases. The fact that intrinsic apoptosis is less triggered in CBMO is of particular importance, since monocytes are generally described to show high susceptibility to caspase-dependent apoptosis [57–59]. This finding is also supported by the cytochrome c release which is—at least partially—blocked by AREG. The diminished cytochrome c release in CBMO can be explained by the increased sAREG levels: AREG functions as an antiapoptotic stimulus in infected CBMO. We showed here comparable antiapoptotic effects of EGF. But in contrast to enhanced levels of AREG in CBMO, EGF levels were reported to be lower in newborns [60]. The stimulus is blocked when inhibiting EGFR, leading to an increase in cleaved caspase levels, which mediate apoptosis. Since PBMO display distinctly decreased sAREG levels after infection, these cells are not exposed to the antiapoptotic stimulus, resulting in higher cleaved caspase levels compared to CBMO, which are not increasable by EGFR inhibition.

In general, a loss of cell viability effectively terminates the acute-phase inflammatory response, thereby characterizing monocyte PICD an important event during inflammation since it balances the underlying pro- and anti-inflammatory processes [11]. Older children and adults are usually able to terminate the inflammation in response to bacterial infections, whereas this counter-reaction often fails in neonates [61]. The failure of terminating CBMO survival prolongs immune competence on the one hand but extends the proinflammatory immune response on the other hand, while evidences suggest that an exaggerated release of cytokines has detrimental sequelae, particularly for neonates [61]. One example are the consequences of sepsis in neonates, which are usually more distinct in clinical symptoms and prognosis compared with that in older children or adults [62]. Our results suggest that AREG might be a key factor in the regulation of PICD and could be a potential candidate to target inflammatory diseases in neonates. Further studies are needed to provide an evidence for its significance *in vivo*.

In summary, this study indicates that AREG inhibits the intrinsic apoptosis pathway through EGFR downstream trafficking as illustrated by the schematic diagram in Figure 6. Since neonatal monocytes show remarkably increased AREG protein levels, it is reasonable to assume that the cytokine is involved in the prevention of PICD in these cells, resulting in an insufficient termination of the inflammatory response. The presented data could contribute to a better understanding of the regulatory elements involved in PICD and could offer a basis for preventive therapeutic strategies to treat neonatal inflammatory diseases.

## Figures and Tables

**(a):** 

PBMO	Nontreated	AREG 0.05 *μ*g/ml	AREG 0.5 *μ*g/ml	*E. coli*	*E. coli* AREG 0.05 *μ*g/ml	*E. coli* AREG 0.05 *μ*g/ml
EGFR^+^ (%)	9.167	10.98	12.38	47.72	52.92	54.02
SD	2.041	1.328	1.909	5.851	4.793	7.529
*p* < 0.05	^∗^			^∗^	^∗^	^∗^

**(b):** 

CBMO	Nontreated	AREG 0.05 *μ*g/ml	AREG 0.5 *μ*g/ml	*E. coli*	*E. coli* AREG 0.05 *μ*g/ml	*E. coli* AREG 0.05 *μ*g/ml
EGFR^+^ (%)	10	10.86	11.62	46.69	47.51	45.07
SD	0.633	0.793	1.951	7.919	3.784	6.669
*p* < 0.05	^∗^			^∗^	^∗^	^∗^

## Data Availability

The data used to support the findings of this study are available from the corresponding author upon request.

## Abbreviations

*α*: Anti
ADAM-17: Disintegrin and metalloproteinase domain-containing protein 17
Akt: Protein kinase B
Apaf-1: Apoptotic protease activating factor 1
APC: Allophycocyanin
AREG: Amphiregulin
BAD: Bcl-2-antagonist of cell death
Bak: Bcl-2-antagonist/killer
Bax: Bcl-2-associated X
Bcl-2: B-cell lymphoma 2
Bcl-X_L_: B-cell lymphoma-extra large
BPD: Bronchopulmonary dysplasia
BSA: Bovine serum albumin
Cas-3: Caspase-3
Cas-9: Caspase-9
CBMC: Cord blood mononuclear cells
CBMO: CD14-positive peripheral blood monocytes from cord blood
DMSO: Dimethyl sulfoxide
dsRed: Recombinant red fluorescent protein
*E. coli*: *Escherichia coli*
EGF: Epidermal growth factor
EGFR: Epidermal growth factor receptor
ELISA: Enzyme-linked immunosorbent assay
ERK: Extracellular signal-regulated kinase
ERK1/2: Extracellular signal-regulated kinases 1/2
FBS: Fetal bovine serum
FITC: Fluorescein isothiocyanate
FSC: Forward scatter
IgG: Immunoglobulin G
IL-6: Interleukin-6
IU: International unit
JNK: C-Jun terminal kinase
MAC: Mitochondrial apoptosis-induced channel
MAPK: Mitogen-activated protein kinase
MFI: Mean fluorescence index
MOI: Multiplicity of infection
NEC: Necrotizing enterocolitis
p38: p38 mitogen-activated kinase
p90^RSK^: 90 kDa ribosomal protein S6 kinase 1
pAkt: Phosphorylated protein kinase B
PARP: Poly (ADP-ribose) polymerase
pBAD: Phosphorylated Bcl-2-antagonist of cell death
PBMC: Peripheral blood mononuclear cells
PBMO: CD14-positive peripheral blood monocytes from adults
PBS: Phosphate-buffered saline
PBS-T: Phosphate-buffered saline Triton-X 100-supplemented
PE: Allophycocyanin
pERK1/2: Phosphorylated extracellular signal-regulated kinases 1/2
PI: Propidium iodide
PI3K: Phosphoinositide 3-kinase
PICD: Phagocytosis-induced cell death
PVL: Periventricular leukomalacia
RT: Room temperature
sAREG: Soluble amphiregulin
SD: Standard deviation
SGA: Small for gestational age
SSC: Sideward scatter
TACE: Tumor necrosis factor *α*-converting enzyme.

## Supplementary Materials

Supplementary Materials Supplementary Figure 1: examples of immunoblots used for quantification of MAPK activation. Blots represent at least two of three independent samples. MAP kinases are given in the figure. Note that the ERK MAP kinase blot shows an additional sample.
