# Supplementary material for: Amphiregulin Regulates Phagocytosis-Induced Cell Death in Monocytes via EGFR and the Bcl-2 Protein Family
Source: Mediators Inflamm. 2019 Nov 3;2019:1603131. doi: 10.1155/2019/1603131 (PMC7012211; doi:10.1155/2019/1603131)
Supplement: Supplementary Materials — Supplementary Figure 1: examples of immunoblots used for quantification of MAPK activation. Blots represent at least two of three independent samples. MAP kinases are given in the figure. Note that the ERK MAP kinase blot shows an additional sample. [file 1603131.f1.docx]

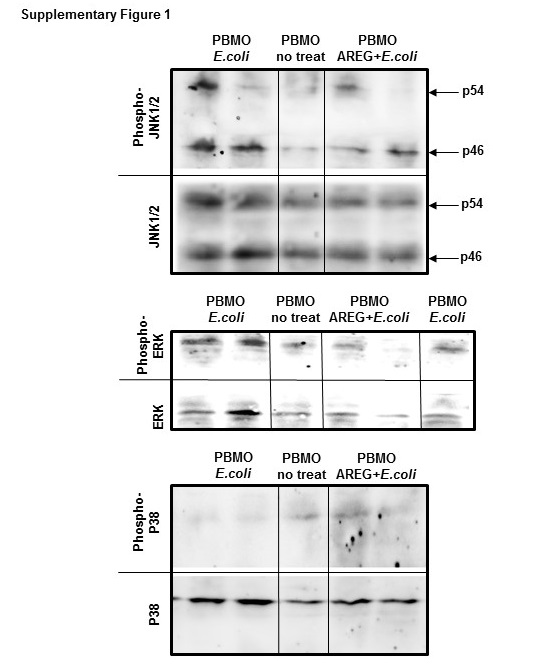


**Supplementary Figure 1: Examples of Immunoblots used for quantification of MAPK activation.** Blots represent at least two of three independent samples. MAP Kinases are given in the figure. Note, that ERK MAP kinase blot shows an additional sample.
